# Asymmetric Programming: A Highly Reliable Metadata Allocation Strategy for MLC NAND Flash Memory-Based Sensor Systems

**DOI:** 10.3390/s141018851

**Published:** 2014-10-10

**Authors:** Min Huang, Zhaoqing Liu, Liyan Qiao

**Affiliations:** Department of Automatic Test and Control, Harbin Institute of Technology, 3033#, Science Park of Harbin Institute of Technology, No.2, Yikuang Street, NanGang District, Harbin 150080, China; E-Mail: jimmyhuanghit@gmail.com

**Keywords:** NAND flash memory, reliability, metadata, MSB page

## Abstract

While the NAND flash memory is widely used as the storage medium in modern sensor systems, the aggressive shrinking of process geometry and an increase in the number of bits stored in each memory cell will inevitably degrade the reliability of NAND flash memory. In particular, it's critical to enhance metadata reliability, which occupies only a small portion of the storage space, but maintains the critical information of the file system and the address translations of the storage system. Metadata damage will cause the system to crash or a large amount of data to be lost. This paper presents Asymmetric Programming, a highly reliable metadata allocation strategy for MLC NAND flash memory storage systems. Our technique exploits for the first time the property of the multi-page architecture of MLC NAND flash memory to improve the reliability of metadata. The basic idea is to keep metadata in most significant bit (MSB) pages which are more reliable than least significant bit (LSB) pages. Thus, we can achieve relatively low bit error rates for metadata. Based on this idea, we propose two strategies to optimize address mapping and garbage collection. We have implemented Asymmetric Programming on a real hardware platform. The experimental results show that Asymmetric Programming can achieve a reduction in the number of page errors of up to 99.05% with the baseline error correction scheme.

## 1. Introduction

Because of the increasing scale and complexity of sensor systems, large amounts of data are being collected, such as medical records, bioinformatics and aircraft health information. It is becoming increasingly more imperative to ensure the fast and safe storage of these data. NAND flash, a semiconductor-based nonvolatile memory, features non-volatility, low power-consumption, fast access time and shock-resistance and therefore is widely used in sensor systems, such as wireless sensor networks.

There are mainly two kinds of NAND flash memory cells, namely single-level cell (SLC) and multi-level cell (MLC) flash memory. MLC NAND flash devices can cost less and have better storage density than SLC NAND flash devices. Therefore, MLC flash devices have been widely adopted by the market, and have become the mainstream flash storage media. Dramatic improvements in the price and capacity of MLC NAND flash memory have been achieved by technological advances, such as the aggressive shrinking of process geometry and an increase in the number of bits stored in each memory cell. These technological advances inevitably degrade the reliability of MLC NAND flash memory.

On the issues of enhancing the reliability of NAND flash based storage systems, most existing works have focused on modifying the components in the current NAND flash memory architecture, e.g., file systems [1], the design of the flash translation layer (FTL) [2–7], and the physical address mapping [8–10]. Some other works suggest that error prevention techniques be implemented to reduce the bit error rate of data stored in NAND flash memory [11–14]. Although these approaches are effective at enhancing the reliability of NAND flash memory, they are not designed for the metadata, which can directly affect the reliability of the whole storage system.

The most effective way of enhancing the reliability of MLC NAND flash memory is to ensure the reliability of metadata [15,16], which occupies only a small portion of the total storage space but maintains the critical information of the file system (*i.e.*, file allocation table and the root folder) and the address translations of the storage system. Therefore, how to enhance the reliability of the metadata stored in NAND flash memory has become an important issue.

Previous studies on the reliability of the metadata in NAND flash memory have normally used a stronger Error Correcting Code (ECC) or redundancy [17–19] to provide extra protection for metadata. Normally, the redundancy methods back up the critical pages containing metadata, and stronger ECC methods provide extra levels of protection for metadata using either software ECC or hardware ECC. They could introduce extra numbers of I/O operations and incur garbage collection overhead. As illustrated in our experimental results, simply applying redundancy to metadata may negatively impact the block erase counts.

Recent technology in MLC NAND flash memory uses multi-page architecture [20]. The different allocation of the data in Least Significant Bit (LSB) pages and Most Significant Bit (MSB) pages in this architecture will directly affect the reliability of the corresponding physical page. This paper for the first time exploits this intrinsic feature of MLC NAND flash to improve the reliability of metadata in MLC NAND flash storage systems. We present Asymmetric Programming, a reliability enhancement strategy that tries to keep all of the metadata in MSB pages following an asymmetrical programming manner, and aims to achieve a relatively low error. By doing this, the error rate for pages containing metadata can be significantly reduced, thereby enhancing the reliability of the entire MLC NAND flash storage system. We propose two strategies, *MSB allocation* and *asymmetric garbage collection*, to reduce the error rate and improve the utilization of physical blocks. Our strategies can effectively enhance the reliability of MLC NAND flash memory while incurring no space overhead and negligible I/O overhead. In addition, the proposed Asymmetric Programming scheme could supplement both ECC and redundancy methods to further improve the reliability of the metadata in MLC NAND flash storage systems.

We have conducted a set of experiments on a real hardware platform. We have designed the MLC NAND flash memory array and implemented the proposed Asymmetric Programming inside the processing system of the Xilinx Zynq on-chip programmable SoC architecture [21]. Asymmetric Programming is compared with a representative design [17] and the default error correction scheme in the Linux kernel [22]. The experimental results show that, Asymmetric Programming can reduce uncorrectable page errors by 88.11% and 72.32% on average compared to [22] and [17], respectively. In terms of I/O performance, Asymmetric Programming only incurs a time overhead of less than 0.07% compared to the baseline error correction scheme.

## 2. Background

### 2.1. Flash Translation Layer and File System

Different from hard disk drives that allow “in-place-update” operation, the physical blocks in NAND flash memory must be erased before they are programmed. Therefore, a software module called the flash translation layer (FTL) is used in NAND flash memory to handle this constraint. FTL maintains the address mapping table [5,23–26], conducts garbage collection operations [27], manages the bad blocks [28], and balances the wear leveling [29] of NAND flash memory.

According to the granularity of address mappings, there are mainly three types of FTL schemes: page-level FTL, block-level FTL, and hybrid-level FTL. Most of the current FTL designs are based on SLC NAND flash memory storage systems. Block-level FTL and some of the hybrid-level FTLs use the block offset to locate the pages within a block, and the pages may be programmed randomly within a block [30], but for MLC NAND flash memory, programming physical pages with the “out-of-order” pattern is prohibited [30]. Therefore, some of these FTLs cannot be applied to MLC NAND flash memory storage systems directly. Since our technique adopts the page-level FTL, it can be applied to MLC NAND flash memory to enhance its reliability.

Several file systems have been specifically designed for NAND flash memory, such as JFFS/JFFS2 [31], and YAFFS/YAFFS2 [32]. Typically, these file systems are dedicated flash file systems and are aimed at embedded applications. They also achieve some functions of FTL (*i.e.*, wear leveling and bad block management). For large-scale storage applications, many general-purpose file systems, such as NTFS [33], FAT [34], and XFS [35], are used without considering the constraints of NAND flash memory. When these file systems are used in the NAND flash memory storage system, additional FTL schemes must be implemented to conceal the special characteristics of NAND flash memory.

### 2.2. Bit Error of MLC NAND Flash

**Figure 1:**
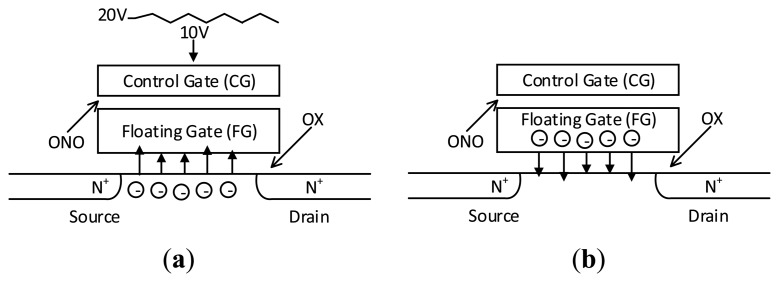
(**a**) Program disturbances cause the bit errors in a NAND flash memory cell. (**b**) Leakages of current causes the bit errors in a NAND flash memory cell.

Bit errors in MLC NAND flash memory are mainly caused by program disturbances and leakage of current (also called retention errors). When programming one selected physical page, a high voltage such as 20 Volts must be applied to the word line (WL) of the target page. This high voltage will then cause disturbances on other unselected pages. An illustration of this process is given in Figure 1a. These disturbances will cause some electrons to be injected into the floating gate (FG) from the base. As a result, the reference voltage of the current will change and the information stored in this cell could be altered.

On the other hand, the conductivity of the oxide may be higher as the P/E cycle increases or the temperature rises. The electrons stored in the FG will leak into the base [36], as shown in Figure 1b. This will also change the reference voltage of the FG and damage the stored information. The leaked current can be calculated using Equations (1) and (2) [20], where A, B and *V_thi_* are constants; *E* is the electric field intensity of the Control Gate (CG); *E_ox_* is the electric field intensity of the oxide; *C_ono_* and *C_ox_* represent the capacitance of ONO (*S_i_O_2_*−*S_i3_N_4_*−*S_i_O_2_*) and the oxide, respectively; *V_th_* is the reference voltage of the cell and *T_ox_* is the thickness of the oxide. Through this model we can observe that the lower the reference voltage of the flash memory cell, the less current will leak. The BER of this cell also would be lower:

(1) $$I_{\text{leak}}=A\cdot E^{2}\cdot \exp(-B/E_{ox})$$

(2) $$E_{ox}=\{C_{\text{ono}}/(C_{ox}+C_{\text{ono}})\}\cdot (V_{th}-V_{\text{thi}})/T_{ox}$$

### 2.3. Multi-Page Architecture of MLC NAND Flash

**Figure 2:**
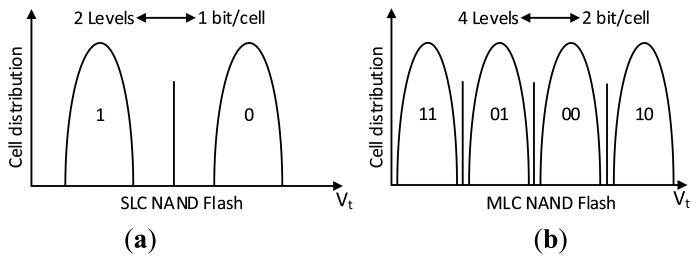
SLC and MLC NAND flash coding and the corresponding reference voltage.

MLC NAND flash memory adopts a multi-page architecture [20] to reduce program disturbances and improve the program speed at the same time. Under this multipage architecture, every two pages form a pair of programmable pages called shared pages. These two pages are further defined as the Least Significant Bit (LSB) and the Most Significant Bit (MSB) pages. For a pair of shared pages, allocation of the data in LSB or MSB pages determines the distortion level of the MLC NAND flash which can directly affect the reliability of the corresponding physical page. Normally, the cell of MLC NAND flash is firstly programmed into two low voltage states, then other two voltage states which represent the information of the MSB page are generated by the second programming process as shown in Figure 2b. Therefore, the MSB page has much lower program distortion as that of the LSB page and much higher reliability because it can directly avoid distortions induced by the first step of the programming process.

## 3. Motivation

**Figure 3.**
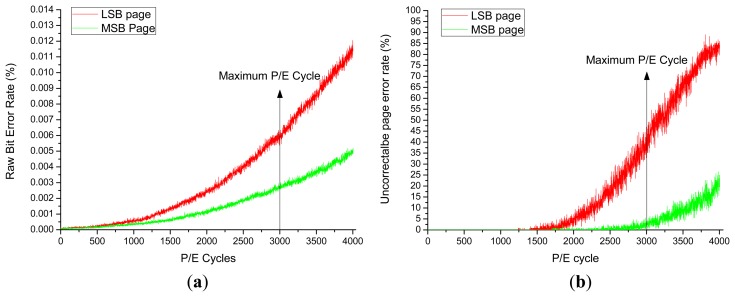
RBER (Raw Bit Error Rate) and uncorrectable page errors *versus* different P/E cycles.

ECC is widely used to reduce the bit error rate of NAND flash memory devices. Combining ECC with the different location of the data, data of the MSB pages has much lower error rates. We tested the raw bit error and the effect of ECC using Micron's MLC flash package 29F64GCBAAA with capacity of 64 Gb and a maximum of 3000 P/E cycles. The results are shown in Figure 3. The raw bit error rate for two different locations of the data is compared in Figure 3a. In this experiment, we program both of LSB pages and MSB pages in ten blocks and analyze the raw bit error rate of the LSB page and the MSB page, respectively. It can be observed that the data allocation has great impact on the raw bit error rate, and the data of MSB pages can achieve a much lower level raw bit error rate than the data of LSB pages. By combining this with ECC, the uncorrectable page error rate can be further reduced (by up to 98.10% of page errors) as shown in Figure 3b. At the time of the maximum 3000 P/E cycles of the lifetime of the tested MLC NAND flash and using 3-bit error correctable BCH code, page errors of over 50% were incurred for the LSB pages, while a reasonably low error rate was maintained by allocating data into MSB pages even after the lifetime of the MLC NAND flash.

Given an N-bit physical page, let the raw bit error rate be R, and the ECC scheme can correct E bits of errors. Then, the probability that there will be more than E faulty bits on a page is [37]:

(3) $$1-\sum_{i=0}^{E}\left(\begin{array}{l} N\\ i \end{array}\right)\cdot {\left(1-R\right)}^{i}\cdot R^{N-i}$$

The bit error rate of one MSB pages is σ*R, where *σ* denotes the ratio of reducing errors of the date in MSB pages than that of LSB pages. For example, for the case in Figure 3, *σ* is 0.5. Then, the probability that there will be more than E faulty bits on a page, or the uncorrectable page error, is:

(4) $$1-\sum_{i=0}^{E}\left(\begin{array}{l} N\\ i \end{array}\right)\cdot {\left(1-\sigma *R\right)}^{i}\cdot {\left(\sigma *R\right)}^{N-i}$$

Based on the above equations, we can calculate the uncorrectable page error rate for LSB pages and MSB pages. The results are presented in Table 1. Compared to date of the LSB pages, allocating data into MSB pages can significantly reduce the uncorrectable page error rate. If we allocate all of the metadata into MSB pages, instead of allocating it into both LSB pages and MSB pages randomly and symmetrically, the metadata will achieve a much lower uncorrectable page error rate following this asymmetric programming manner. Then the reliability of the whole system will be improved. Also, different from redundancy methods which will induce extra space overhead and eventually harm both the I/O performance and endurance of NAN flash memory, LSB pages is still available when allocating the metadata into MSB pages, which means that there will be no space overhead. This observation motives us to present a fault-tolerant scheme to provide a more reliable MLC NAND flash memory storage system.

## 4. Asymmetric Programming: A Reliability Enhancement Scheme for Metadata

### 4.1. Framework

**Figure 4:**
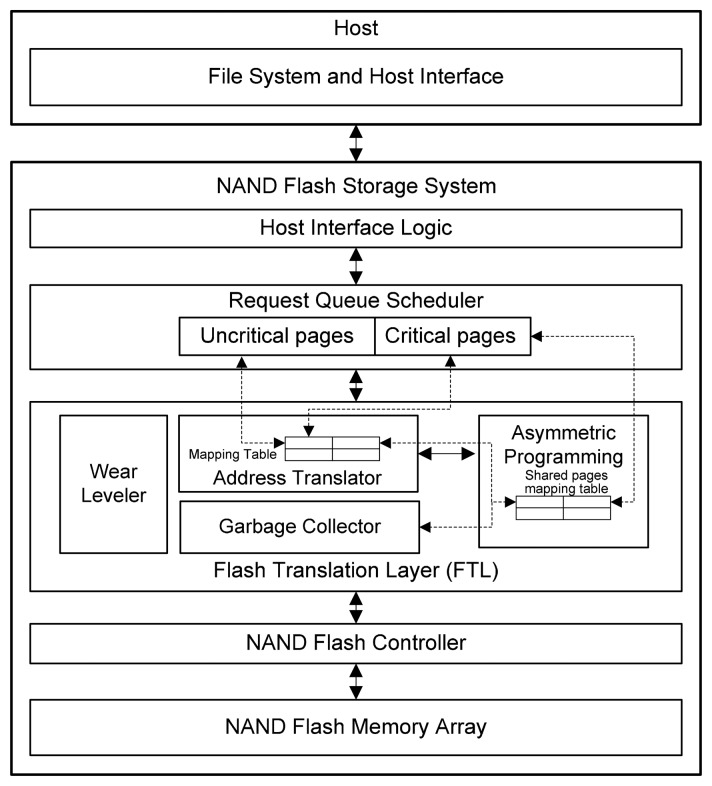
System architecture of Asymmetric Programming.

Asymmetric Programming is an address mapping strategy that introduces transparent management between the file system and hardware to protect the metadata from errors. The system architecture of Asymmetric Programming is shown in Figure 4. In the architecture, the host system and the storage system are connected through the host interface. Most widely used host interfaces, such as SATA II and USB (2.0 or 3.0 version), support outstanding requests. The storage system can serve multiple “out-of-order” requests and form a queue. The request queue scheduler is utilized to manage the queue to optimize the I/O performance by scheduling the requests in the queue.

In MLC NAND flash memory storage systems, physical pages must be programmed sequentially from the least significant page address to the most significant page address. Programming pages with the “out-of-order” pattern is prohibited [30]. To handle this constraint, we present Asymmetric Programming, which works in the flash translation layer (FTL) and reorganizes the address mappings. The objective is to enhance the reliability of MLC NAND flash memory by providing a more enhanced metadata protection mechanism.

Asymmetric Programming adopts two reliability techniques, namely *MSB allocation* and *asymmetric garbage collection*, to enhance the reliability of metadata in MLC NAND flash memory storage systems. The MSB allocation is a reliability strategy that identifies the pages that contain metadata and allocates metadata pages to MSB pages that have lower bit error rates. Asymmetric garbage collection is another reliability strategy that aims to fully utilize the storage space when triggering garbage collection operations. This strategy uses a small buffer to reorganize the valid pages and assign the pages to free pages based on the importance of the data. In the following subsections, we will present these two strategies.

### 4.2. Metadata Identification

Metadata such as file system metadata are normally stored in predefined locations with fixed logical addresses. This characteristic helps us identify the metadata and enhances the reliability of flash. Among the metadata, the file system metadata contributes to the major amount of read and write accesses. File systems normally format the disk and write logical formatting information needed by the file system onto the disk. File system metadata, such as file allocation table and the root folder, must be stored in a fixed location so that the files needed to start the system can be correctly located.

**Figure 5:**
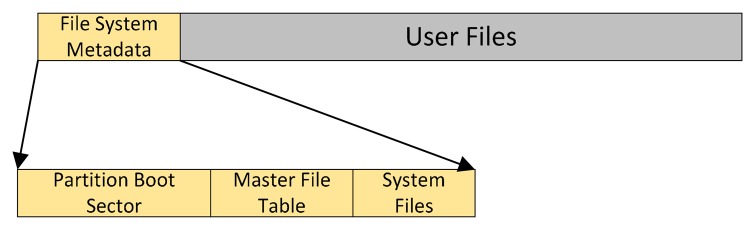
The layout of the NTFS volume.

In modern file systems, such as NTFS, every sector on an NTFS volume that is allocated belongs to some file. The file system metadata is also part of a file. Figure 5 illustrates the layout of an NTFS volume when formatting has finished. The first file on an NTFS volume is the Master File Table (MFT), which contains a set of files maintaining the metadata used to implement the file system structure. In NTFS, the location of MFT is fixed. The NTFS file system reserves the first 16 records in the MFT for the information about these metadata files. The NTFS file system uses about 1 MB for the metadata files and the first 16 records in the MFT.

Besides the file system metadata, the metadata for wear-leveling are used to maintain the block erase counts and to select a victim block for garbage collection in NAND flash memory. Current wear-leveling algorithms normally use heuristics such as priority queue to find the victim block that has the minimum erase counts or has the lowest block utilization. The implementation of these wear-leveling algorithms needs to maintain metadata at the per block level.

NAND flash memory storage also needs to maintain the address mapping table to track the mapping between logical addresses and physical addresses. When writing data into NAND flash memory, the address mapping table will be updated in the RAM and synchronized to NAND flash memory periodically. The tree-like data structure (e.g., B+ tree) has been widely adopted in the FTL schemes to manage the address mapping table. For the tree data structure, the more random writes are, the more indexing nodes have to be updated in the in-memory data structure (e.g., on-flash index in UBIFS). Since the on-chip RAM only has very limited capacity, the metadata stored in RAM has to be frequently flushed from volatile cache to NAND flash memory, which could cause frequent metadata synchronization.

To fully utilize the limited RAM space and improve the write performance, many FTL schemes adopt the demand-based strategy. In demand-based FTL schemes, several physical blocks (called translation blocks) are used to hold the address mapping information that could not be stored in RAM. When running out of translation blocks, the valid pages in translation blocks have to be merged. The victim block that contains all invalid data will be erased. If multiple valid pages are distributed to different translation blocks, this case will trigger many consecutive garbage collection operations. Therefore, the more random the writes are, the more merge operations are triggered. Then the amount of metadata for address mappings is determined by the specific FTL design.

From the above analysis, we found that the logical requests (*i.e.*, logical page numbers) for metadata will be different from those for normal data. They are handled by either file systems or the dedicated FTL schemes. The logical locations that store the metadata have the natural isolations from those of the normal data. File systems or FTL schemes could further manipulate the logical to physical mappings and issue the read or write requests to different physical locations. Therefore, we can utilize this feature and provide extra protections for metadata stored in NAND flash memory. This can significantly enhance the reliability of NAND flash memory storage systems.

### 4.3. MSB Allocation

Our MSB allocation strategy issues write operations based on the content stored in a page. Algorithm 1 describes the process of a write operation. Algorithm 1 has two inputs: a logical page number (LPN) and its content. The algorithm will first distinguish critical pages, which contains metadata of the system, from non-critical pages. If the request is a critical page, the FTL allocates one physical page for the request (line 1). The MSB allocation strategy tries to allocate critical pages to MSB pages. It will look up the shared pages mapping table, which is configured by the chip manufacturer, to check the allocated physical page whether a MSB page or not. To the best of our knowledge, this is the first work that utilizes the shared pages mapping table to achieve the recognition of the LSB page and the MSB page in the FTL.


**Algorithm 1.** Write operation for Asymmetric Programming.
**Input:** A logical page number (*LPN #M*), LPN page content.**Output:** Write the content to one physical page1 FTL allocates a *PPN #N* for *LPN #M*2 **if**
*LPN #M* is a critical page **then**3  Query the shared pages mapping table to check if *PPN #N* is MSB page4  **if**
*PPN #N* is MSB page **then**5  Write *LPN #M* into *PPN #N*6  **else**7   Invalidate the mapping <*LPN #M, PPN #N*>8   Send a signal to Request Queue Scheduler to delay the current request9   **if** there's no uncritical page request **then**10   Find the nearest MSB page11    Invalidate all the pages between the current page and the nearest MSB physical page12   **end if**13  **end if**14  **Finish**15 **end if**16 Update the mapping table17 Write the content into NAND flash


**Figure 6.**
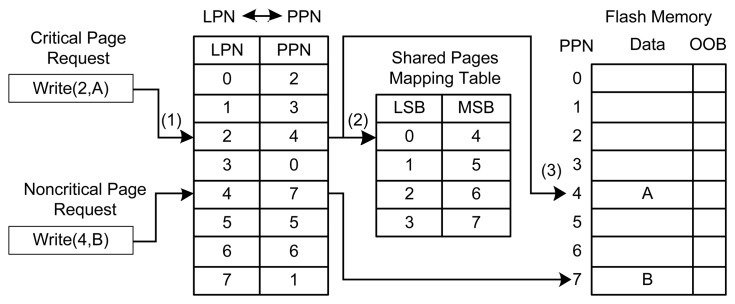
An example of the MSB allocation strategy of Asymmetric Programming.

If the page does not contain metadata (lines 16–17), the MSB allocation strategy will bypass the shared pages mapping table. Such write requests will be directly issued to available free pages in the sequential manner of programming. Both the LSB and MSB pages can be utilized to store normal data. Figure 6 shows an example of the MSB allocation strategy. For the write request (LPN #2, A), where A is the content to be written in the NAND flash and LPN #2 belongs to a critical page, the MSB allocation strategy will search the logical page number to the physical page number (PPN) addressing mapping table maintained by the FTL and find the corresponding PPN (PPN #4). It will further check the shared pages mapping table provided by the chip manufacturer. This table indicates that PPN #4 belongs to MSB pages. The MSB allocation strategy will then write content A to the MSB page. For the write request (LPN #4, B), which denotes a non-critical page, our strategy will bypass the search operation in the shared pages mapping table and issue a write operation to the PPN provided by the FTL (*i.e.*, PPN #7).

If the FTL assigns an LSB page to a critical page, the MSB allocation strategy will send a signal to the Request Queue Scheduler to delay this request. The next uncritical page write request will be kicked out to fit this LSB page. Then the Request Queue Scheduler will serve the delayed request again and repeat the above operations to allocate a MSB physical page for the critical write request. This procedure is called a request feedback operation. If no non-critical page is available in the request queue (lines 9–12), the next available MSB page will be found directly. The pages between the current page and the next available MSB page will be marked as invalid. In this way, the MSB strategy can guarantee that the next critical page will be served by an MSB page.

**Figure 7:**
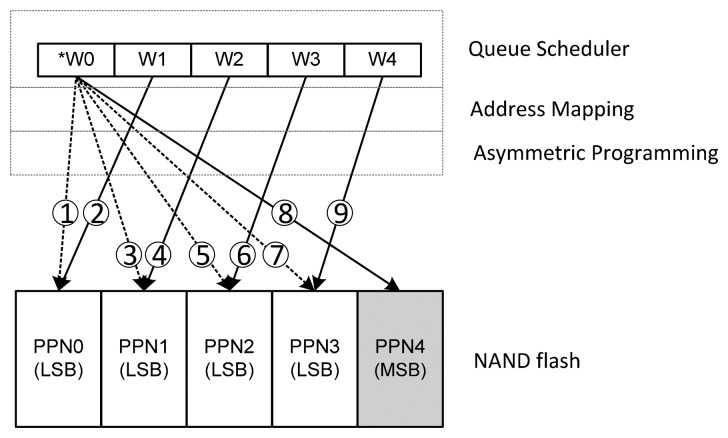
An example of the request feedback operation.

Figure 7 shows an example of request feedback operation where W0 is a critical page write request and W1-4 are three uncritical page requests. Pure page-level FTL is adopted in this example. In the first step, the FTL allocate PPN0 for the request W0. But as W0 is a critical page request and PPN0 is a LSB physical page, our asymmetric programming strategy invalidates this mapping, and delays W0. Then it will allocate W1 which is an uncritical page request to PPN0. This process is a request feedback operation. After that, the FTL allocates PPN1 for the critical page request W0, but still, the PPN1 is a LSB physical page, then W0 is delayed and the FTL writes W2 into PPN2. The feedback operation is conducted until the FTL allocates PPN4 which is a physical MSB page for W0. Then the process of MSB allocation strategy is done.

### 4.4. Asymmetric Garbage Collection

In NAND flash memory storage systems, a garbage collection operation normally includes the following three steps:

- Step 1: The garbage collector picks up the victim block;
- Step 2: The garbage collector copies the valid pages of the victim block into a free block;
- Step 3: The garbage collector erases the victim block and adds the victim block into the free block pool.

**Algorithm 2.** Valid page copies in garbage collection for Asymmetric Programming.
**Input:** Valid pages in the valid page buffer. *N_p_*: The count of physical pages in one physical block.**Output:** Write the valid pages in the valid page buffer into a free physical block1 **for**
*i* = *0; i* < *N_p_, i*++ **do**2 **if**
*PPN #L (the Physical Page Number of page i)* is LSB page **then**3 Fetch one uncritical page (*LPN #K*) from the valid page buffer4 **if** there's no uncritical page in the valid page buffer **then**5    Do nothing.6   **else**7    Write *LPN #K* into *PPN #L*8    Kick *LPN #K* out of the valid page buffer9    Update the mapping table10   **end if**11   **else**12  **if**
*PPN #L (the Physical Page Number of page i)* is MSB page **then**13   Fetch one critical page (*LPN #Q*) from the valid page buffer14   **if** there's no critical page in the valid page buffer **then**15  Fetch one uncritical page (*LPN #K*) from the valid page buffer16 **if** there's no uncritical page in the valid page buffer **then**17    Do nothing.18   **else**19    Write *LPN #K* into *PPN #L*20    Kick *LPN #K* out of the valid page buffer21    Update the mapping table22   **end if**23  **else**24    Write *LPN #Q* into *PPN #L*25    Kick *LPN #Q* out of the valid page buffer26    Update the mapping table27   **end if**28  **end if**29  **if** there's no LPNs in the valid page buffer **then**30  break31 **end if**32 **end for**


Steps 1 and 3 will not influence the location of critical pages in the MLC NAND flash memory. However, when the garbage collector copies valid pages from the victim block to a free block, we need to make sure that the critical pages are still stored in the MSB physical pages. To achieve this objective, step 2 of the garbage collection process has to be modified. We propose the asymmetric garbage collection strategy to handle this issue. Our basic idea is to use a small buffer to reorganize write requests. Algorithm 2 presents the basic procedures of the garbage collection operations in Asymmetric Programming.

In Algorithm 2, a small buffer is used to store all valid pages from the victim block. Our strategy issues write operations for critical pages to the free block. For the LSB page of the free block, the asymmetric garbage collection strategy will look up the valid page buffer and fetch one uncritical page and write it into this physical LSB page (lines 2–10). If the writing process is finished successfully, the fetched uncritical page will be kicked out from the valid page buffer. For the MSB page of the free block, the asymmetric garbage collection strategy will try to fetch one critical page from the valid page buffer and write it into this physical MSB page then kick out the copied critical page out of the valid page buffer (lines 24–27). Thus, critical pages can still be ensured to be stored in physical MSB pages after the garbage collection process. If there's no critical pages left in the valid page buffer, then the asymmetric garbage collection strategy will fetch one uncritical page and copy it into this physical MSB page (lines 14–22). The garbage collection is done when all the valid pages in the valid page buffer are copied into the free block (line 30).

**Figure 8.**
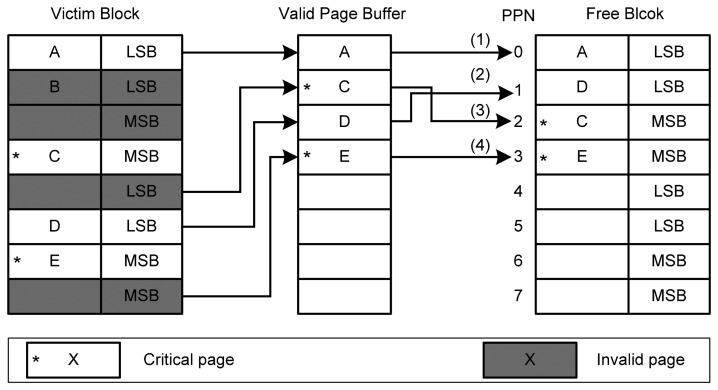
An example of the valid page copy strategy of the asymmetric garbage collection.

We also use an example to illustrate the asymmetric garbage collection strategy. Figure 8 shows the example. For the purpose of illustration, we assume that there are eight pages in a physical block. One physical block is selected as the victim block. In this block, four pages are valid pages, and pages C and E belong to critical pages. These four valid pages will be copied to the buffer. Our strategy will first issue write operations for the uncritical page (*i.e.*, page A). Similarly, another uncritical page will be written to page 1 following the sequential manner of programming in MLC NAND flash memory. For the physical page 2 and 3 which are MSB pages, our strategy will issue write operations for critical pages (*i.e.*, pages C and E).

## 5. Evaluation and Analysis

To evaluate the effectiveness of our proposed Asymmetric Programming scheme, we conducted a series of experiments on a real hardware platform. The objective of the evaluation was to evaluate the approach in terms of three performance metrics: the number of uncorrectable page errors, the I/O performance and the garbage collection efficiency. In this section, we first introduce the experimental environment and performance metrics. We then present the experimental results and the discussion.

### 5.1. Experimental Setup

As shown in Figures 10a and 11, the hardware platform consists of a daughter board and a core board. The core board is built based on Xilinx Zynq on-chip programmable SoC architecture [21], which is equipped with dual hardware ARM Cortex A9 processors running at 800 MHz, 1 GB DDR3 RAM. The daughter board supports up to eight NAND flash packages with the sockets. In this evaluation, we used 64 Gb Micron 29F64GCBAAA NAND flash memory chips. The configuration of this MLC NAND flash is shown in Table 2.

**Figure 9.**
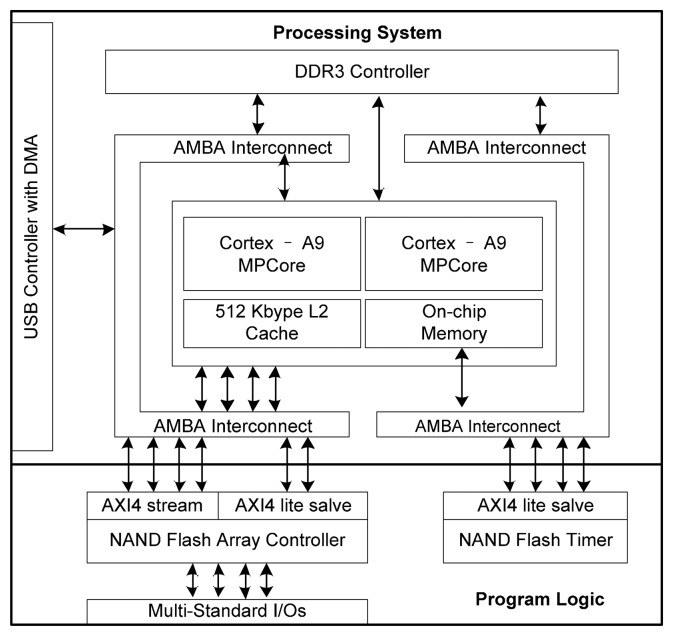
The firmware architecture of the experimental platform.

All of the firmware were implemented in the Zynq SoC. As shown in Figure 9, the Zynq SoC resource is comprised of two parts: the processing system and the programmable logic. The processing system has dual ARM cores and other peripheral controllers, such as USB 2.0 interface controller and a DDR3 controller. The NAND flash controller was implemented in the programmable logic, which is a standard Xilinx 7 series FPGA. The programmable logic connects to the AMBA interconnect through 32-bit high-speed GP AXI port with a bandwidth of up to 1 GB/s. We wrapped the NAND flash controller with one AXI streaming interface which can achieve the maximum bandwidth of the AXI interface. One AXI lite slave interface was used to operate the registers of the NAND flash controller, making it possible to take full advantage of the bandwidth of the high-speed High Performance (HP) AXI port of Zynq.

Due to the limited bandwidth of the USB 2.0 interface, we also designed a hardware NAND flash timer to precisely monitor the response time of NAND flash memory. This hardware NAND flash timer can reach a resolution of 10 ns, which is accurate enough for our time-related experiments. Asymmetric Programming was implemented in the processing system of Xilinx Zynq.

To evaluate Asymmetric Programming, we adopted the NTFS file system on the host computer and connected our hardware platform with the USB interface. To evaluate the efficiency of Asymmetric Programming, we focused on enhancing the reliability of the metadata in the NTFS file system. The size of the NTFS allocation unit size is configured as 8192 bytes and the original metadata were allocated to the first 1024 logical pages (*i.e.*, #LPN 0 to #LPN 1023).

Pure page-level FTL was implemented as a thread in the FreeRTOS which is widely used free real time operating system and ran at the processing system of the hardware platform, which is equipped with dual ARM cores. We maintained the mapping table of the logical page number (LPN) and physical page number (PPN), and the other important data structures (e.g., bad block information and a shared pages mapping table) in the RAM (DDR3). The capacity of DDR3 of our hardware platform is 1 GB, which is big enough to keep these data structures. We modified the widely used greedy policy [38] in the garbage collection process and made the metric of the P/E cycles and the metric of the number of valid pages in the block both have 50% weight to find the victim block. Thus, the wear leveling was also very effective in our design. An additional system monitor module was also added into the FTL, which can record all of the flash operations (e.g., the number of read, program and erase operations, the overall request number and the number of the valid page copies in the garbage collection process).

We compared Asymmetric Programming with a representative Meta-Cure scheme [17] and the default error correction scheme in the Linux kernel (labeled as “Baseline”). First, we use the real workload of the sensor system of Zynq board to evaluate the effectiveness of Asymmetric Programming scheme. Then, we apply IOMeter [39], which is widely used as a standard and configurable workload generator, to conduct more comprehensive experiments to further analyze the proposed scheme.

### 5.2. Experimental Results and Analysis with Zynq On-Board Sensor System

**Figure 10:**
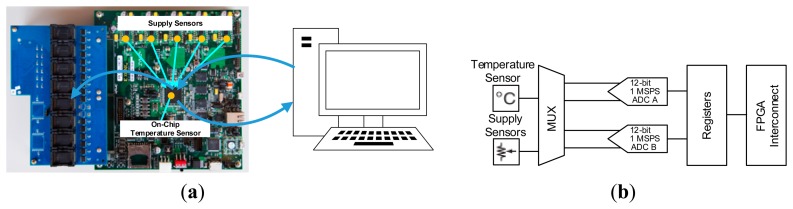
(**a**) The system architecture and illustration of the data flow (**b**) Zynq on-board sensor system block diagram.

In this section, we present the experimental results with an analysis of Asymmetric Programming with Zynq on-board sensor system. The system architecture of the experimental setup is shown in Figure 10a. The Zynq on-board sensor system is a typical sensor system which includes on-board sensors and a dual 12-bit, 1 Mega sample per second (MSPS) Analog to Digital Converter (ADC) as shown in Figure 10b [40]. On-board sensors support measurement of the power supply voltages of 11 channels and die temperature. The ADC conversion data is stored in dedicated registers called status registers. These registers are accessible through the FPGA interconnect, which is an AXI Slave interface in our design. As shown in Figure 10a, the data of Zynq on-board sensors is read out by the computer through the Ethernet interface and the computer then writes the data back into the NAND flash on the daughter card which is configured as the description of Section 5.1. We store the received data into binary files and each file has the size of 16 KB. All of the sensor data is kept in the space of 256 MB of one NAND flash. When the capacity runs out, we just erase the old data and continue to store the incoming data into this area.

As shown in Table 3, the 29F64GCBAAA MLC NAND flash is baked to 2000 P/E cycles first and 4-bit BCH code is applied. The statistic results of the workload gained from Zynq on-board sensor system are also shown in Table 3.

We compared Asymmetric Programming with a representative scheme Meta-Cure [17] and the default error correction scheme in the Linux kernel (labeled as “Baseline”) in terms of three performance metrics: the number of uncorrectable page errors, the I/O performance and the garbage collection efficiency. The experimental results are shown in Table 4.

Compared with the Baseline scheme, we can observe that the proposed Asymmetric Programming can reduce up to 91.97% uncorrectable page errors while only inducing 0.04% and 0.23% overhead in terms of average response time and number of block erase counts, respectively. The additional block erase operations of Asymmetric Programming are caused by the request feedback operations, but its overhead is negligible compared with that of Meta-Cure. This further proves the effectiveness of Algorithm 1. The overhead of extra block erase operations eventually results in the average response time overhead, but it is still negligible according to Table 4.

Since Asymmetric Programming enhances the reliability of data directly in the programming process and allocation process and does not require extra program operations like Meta-Cure, therefore, the proposed scheme has much less garbage collection operations. Asymmetric Programming also does not need to read backup pages to search for the correctable page. Thus, it also has less read operations than Meta-Cure. Based on these analysis, it is reasonable for Asymmetric Programming to increase the I/O performance by 9.24% compared with Meta-Cure.

Thus, we can conclude that the proposed Asymmetric Programming can achieve higher reliability than Meta-Cure, while induce negligible overheads in terms of the I/O performance and the garbage collection efficiency in a Zynq on-board sensor system.

### 5.3. Experimental Results and Analysis with IOMeter Tool

**Figure 11.**
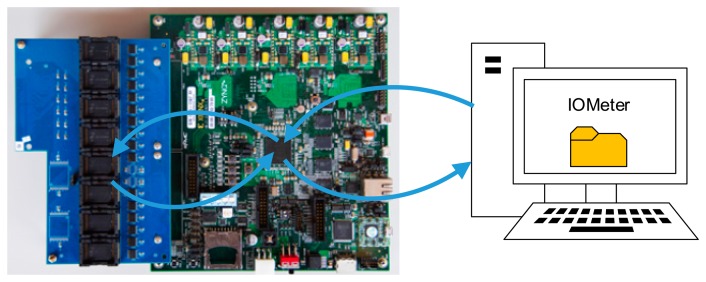
The system architecture and illustration of the data flow with IOMeter

To fully evaluate the proposed Asymmetric Programming and measure its performance with more comprehensive metrics, we conducted more experiments with IOMeter [39]. The system architecture is shown in Figure 11. IOMeter is widely used as a standard and configurable workload generator and it conducts the write and read operations to the experimental platform according to the configuration.

In this section, we present the experimental results with an analysis with IOMeter. We also compared Asymmetric Programming with a representative scheme Meta-Cure [17] and the default error correction scheme in the Linux kernel (labeled as “Baseline”) in terms of three performance metrics: the number of uncorrectable page errors, the I/O performance and the garbage collection efficiency. We also use 256 MB space in one NAND flash package to conduct the experiment and the allocation unit size of NTFS is set as 8192 bytes. Thus, the size of each read or write request of the NTFS is the integer multiples of the allocation unit size and is aligned to the size of physical page which is also 8192 bytes.

#### 5.3.1. Uncorrectable Page Errors

As the program and erase cycles (P/E cycles) increase, the bit error rate (BER) increases correspondingly. Table 5 presents the number of uncorrectable page errors under two levels of wear, which are 1000 and 3000 P/E cycles. The objective is to quantify the error correction capability beyond the built-in ECC when the NAND flash is in a normal condition and a nearly worn out condition. The maximum number of P/E cycles of the NAND flash chip used in the experiment was 3000. In Table 5, “ECC strength” indicates the number of bits of errors that can be corrected by the BCH code for one physical page. The workload generated by the IOMeter was configured with 50% random requests and 50% read accesses and lasted for 10 minutes.

From the experimental results, it can be seen that the number of uncorrectable pages decreases dramatically as ECC strength increases. Although Meta-Cure can reduce the number of error pages significantly, our proposed Asymmetric Programming can further improve the performance and cause the error rate to be maintained at a very low level. When the P/E cycles are 1000 times, compared with the baseline scheme, Asymmetric Programming can avoid up to 40.75% of page errors even without any ECC (0-bit ECC).

If Asymmetric Programming is combined with the BCH code, reduction in page errors of at least 77.93% can be achieved. Compared with Meta-Cure, our proposed Asymmetric Programming can reduce page errors by 72.32% on average, which means that Asymmetric Programming can improve the reliability of system critical page by 72.32% compared to Meta-Cure. When the P/E cycles are 3000, the NAND flash is on the verge of wearing out. The number of uncorrectable pages increases sharply as shown in Table 5. However, Asymmetric Programming can still reduce error pages by up to 94.17%. Compared to the baseline scheme and Meta-Cure, Asymmetric Programming can also reduce the number of uncorrectable page errors by an average of 77.69% and 56.67%, respectively. These results show that Asymmetric Programming can work effectively even if the NAND flash is approaching its maximum lifetime. Through the behavior of Asymmetric Programming under the two levels of wears, we can conclude that Asymmetric Programming effectively enhances the reliability of critical pages during the whole lifetime of NAND flash memory.

#### 5.3.2. I/O Performance

I/O performance is another important performance metric for evaluating the NAND flash memory storage system. We evaluated the I/O performance of the proposed scheme in terms of two metrics: the average response time and the response time for read operations. To fully evaluate these time-related cases of Asymmetric Programming, we ran the proposed scheme under workloads with different ratios of random accesses and different percentages of read operations generated by IOMeter. The error correction capability of the BCH code was configured as four bits. To accurately measure the response time of each request, we used the implemented hardware NAND flash timer. The precision of the response time was 10 nanosecond. We recorded the response time of each request and calculated the required time value.

The experimental results for average response time are shown in Table 6 and Table 7. We observe that Asymmetric Programming can achieve similar average response times as the baseline scheme. From the results, it can be seen that the overhead for the average response time ranges between 0.002% and 0.33%. These results show that the I/O performance overhead incurred by Asymmetric Programming is negligible.

From Tables 6 and 7, we also find that the average response time of Asymmetric Programming is not sensitive to the percentage of random accesses but is highly sensitive to the ratio of write operations. That is because the more write operations in the workload, the more frequently garbage collection is needed and the average response time will be longer. For different ratios of random accesses, the number of block erase counts is relatively stable as shown in Figure 14c. Therefore, the average response time will also be relatively stable.

We conducted experiments on performance under different percentages of random accesses with a 50% read operation workload as shown in Table 6. Compared to Meta-Cure, our scheme can improve the average response time by 6.57% on average, while inducing only 0.07% overhead compared with the baseline scheme. This means that our proposed scheme enhances the reliability of the critical pages of storage system with only a negligible average response time overhead.

**Figure 12:**
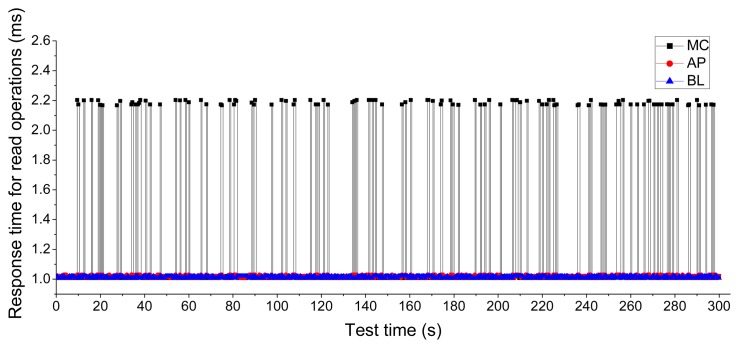
Response time for the read operation of Baseline (BL), Meta-Cure (MC), and Asymmetric Programming (AP).

The response time of the read operation is another important issue. We used a workload with random accesses of 50% and a read operation of 50% to verify the performance of the Asymmetric Programming read operation. The workload lasted for five minutes. The experimental results shown in Figure 12 indicate that Asymmetric Programming can achieve nearly the same response time as the baseline scheme for read operations, while Meta-Cure has a response time over two times longer for read operations. This is because Meta-Cure must read one or more redundant backup pages for critical pages to restore the damaged metadata. That means an overhead cost of two or more times in the response time of read operations. We conducted this experiment using NAND flash with 1000 P/E cycles. As the P/E cycles increase, the uncorrectable page errors will also increase. The response time of Meta-Cure will increase correspondingly. However, Asymmetric Programming improves the reliability of metadata in the programming process without the need to conduct any extra read operations. Therefore, Asymmetric Programming will not cause any overhead in terms of response time for read operations.

#### 5.3.3. Garbage Collection and Endurance

The endurance of NAND flash is one of the most important factors to take into account when analyzing the reliability of flash memory. The endurance of NAND flash is mainly affected by the number of block erase counts in the flash. We also present the total number of write and read operations and the number of valid pages that were copied to further exploit the garbage collection efficiency of Asymmetric Programming. All of the workloads were generated by IOMeter, and each workload lasted for five minutes for each experiment.

**Figure 13:**
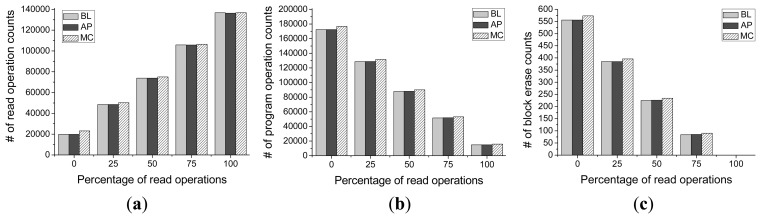
(**a**) The total number of read operations (**b**) The total number of write operations (**c**) The number of block erase counts in Asymmetric Programming (AP) with different percentages of read operations.

**Figure 14:**
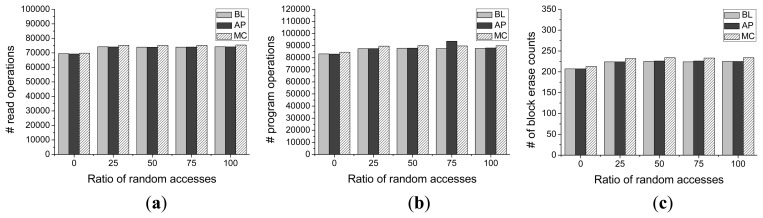
(**a**) The total number of read operations (**b**) The total number of write operations (**c**) The number of block erase counts in Asymmetric Programming (AP) with different ratios of random accesses.

Figures 13 and 14 present the number of block erase counts and the total number of write and read operations with different percentages of read operations and different ratios of random accesses. From the results of Figures 13c and 14c, we observe that Asymmetric Programming can achieve almost the same number of block erase counts as the baseline scheme, which means that Asymmetric Programming will not induce overhead for the endurance of the NAND flash. This result also indicates that our proposed write algorithm (Algorithm 1) is very effective at improving block utilization and that it inserts most of the uncritical page data into the physical pages between the adjacent MSB pages by conducting request feedback operations using request queue scheduler.

Compared with Figure 14c, we can see that the number of block erase counts is affected by the number of write operations for the baseline scheme, Meta-Cure, and Asymmetric Programming schemes. Because of the intrinsic feature of IOMeter, to meet the configuration of the random ratio, it divides the workload into several segments and every segment has accurate random request ratio according the configuration. There are 256 pages per block in the flash package we used in the experiments, which is larger than the segment size of the workload of IOMeter. Thus, except for all sequential accesses (0% random accesses), other different ratios of random accesses would be seemed as all random accesses to the flash package we use. Therefore, the erase count of different ratios of random accesses will not be the same except for all sequential accesses.

The total number of read operations and that of write operations are shown in Figure 13a,b, respectively. Compared with Figure 13c, we can clearly see that the number of block erase counts is affected by the number of write operations for the baseline scheme, Meta-Cure, and Asymmetric Programming schemes. In addition, the total number of read and write operation of the proposed scheme is still the same as that of baseline scheme and much smaller than that of Meta-Cure scheme. This result indicates that Asymmetric Programming scheme induces very few extra write operations when conducting request feedback operation. Also, by combining the results in Figure 14a–c, we can see that none of these three schemes are sensitive to the percentage of read/write operations.

**Figure 15:**
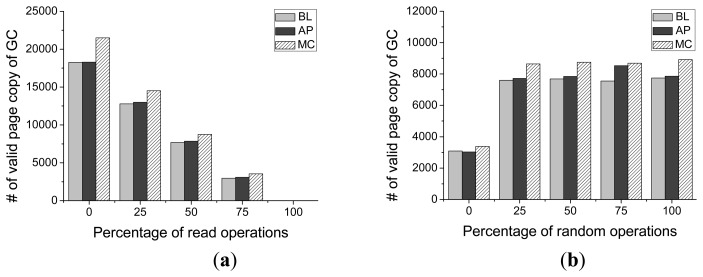
The number of valid page copy operations for Baseline (BL), Meta-Cure (MC), and Asymmetric Programming (AP) during garbage collection operations with different (**a**) Percentages of read operations (**b**) Ratios of random accesses.

The number of valid page copies is another important factor in evaluating the efficiency of the garbage collection process. Figure 15a,b present the number of valid page copy operations with different percentages of read operations and different ratios of random accesses for the baseline, Meta-Cure and Asymmetric Programming, respectively. We can see that Asymmetric Programming achieves nearly similar numbers of valid page copy operations as that of the baseline scheme with different percentages of read operation and the case with different ratios of random accesses. Compared with the Meta-Cure scheme, Asymmetric Programming and Meta-Cure have much smaller numbers of valid page copy operations. This is because there are few extra garbage collection operations caused by the Asymmetric Programming scheme. This result further proves that the proposed Asymmetric Programming scheme has much higher garbage collection efficiency and better endurance than that of Meta-Cure scheme.

## 6. Discussion and Analysis

In this section, we present the discussion about the proposed Asymmetric Programming scheme in terms of performance analysis and the induced overhead.

### 6.1. Performance Analysis

We present a performance analysis of Asymmetric Programming in terms of read performance write performance and garbage collection efficiency.

(1) *Impact on Read Performance:* When the file system conducts reading operations, the read request will query the mapping table of FTL to get the physical address of the corresponding logical address of the request. Then, the NAND flash controller will return the data of the physical page. Asymmetric Programming does not need to modify the FTL address mapping table or add any extra mappings for the read process. Therefore, Asymmetric Programming will not bring any overhead to the read performance as shown in results of Figure 12.
(2) *Impact on Write Performance:* When performing write operations, Asymmetric Programming will directly bypass the uncritical page write requests, which make up the greater part of the I/O requests. This will not incur overhead for write performance of uncritical pages. For those write requests of critical pages, the time overhead is incurred by the shared pages mapping table searching time, the extra request feedback operations and the garbage collection process. Asymmetric Programming needs to search the shared pages mapping to find the MSB page. The time spent on searching for an entry is set as *Tp*. The searching time is determined by the page number of one block (set as *Np*). The *Np* of commercial NAND flash is usually no more than 256. For example, we used Micron's 29F64GCBAAA NAND flash memory chip which consists of 256 pages in one block. Thus, there are no more than 128 (256/2) entries in the shared pages mapping table. Fetching these entries in the shared pages mapping table will take at most several hundreds of nanoseconds and its impact to the write performance can be negligible.
  As described in Algorithm 1, Asymmetric Programming may also introduce extra request feedback operations if the current critical page request is not allocated in the MSB page. Then there will be extra time overhead for write operations. The time overhead is related to the number of request feedback operations. Because most of the operations of the file systems are file-based operations and metadata only occupies a small part of the file, most of the critical requests are random requests. That means, in most cases, one request feedback operation could be enough for the critical pages to be stored in MSB pages. Therefore, the time overhead introduced by the request feedback operation would be small. This conclusion is well proved by experimental results of Figures 13 and 14.
(3) *Impact on Garbage Collection Performance:* During the garbage collection process, Asymmetric Programming copies all of the victim pages into the buffer and then reallocates the order of all the valid pages. Comparing the normal garbage collection process without Asymmetric Programming, it should be noted that Asymmetric Programming has to search the shared pages mapping table to ensure that all of the valid critical pages are copied into the MSB pages of the target block. Each valid page will add one extra search for the shared pages mapping. The total time cost is Np*Tp. Taking Micron's 29F64GCBAAA as an example, *Np* is 256 and *Tp* is about 300 ns. Thus, the overall time for Asymmetric Programming is about 38 μs.

Assuming that the time required to read a page, program a page, and erase a block are *Trd*, *Tprog*, and *Ters*, respectively, and that half of the pages in the victim block are valid pages, then the overall time cost of valid page copy and block erase operations will be *Np/2* * *(Trd* + *Tprog)* + *Ters*. In typical NAND flash memory chips, *Trd*, *Tprog* and *Ters* are 75 μs, 1300 μs and 3800 μs, respectively. Thus, the time needed for valid copy and block erase operations of one instance of garbage collection is 179,800 μs. This means Asymmetric Programming will have a time overhead of less than 0.1% for the garbage collection process. This conclusion is indicated by the results of Figures 13 and 15, in which the proposed Asymmetric Programming scheme has almost the same garbage collection efficiency in terms of the number of block erase counts and the number of valid page copy operations.

### 6.2. Overhead Analysis

**Figure 16:**
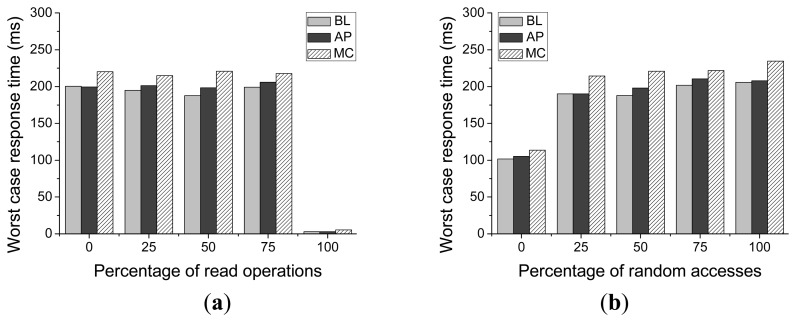
The worst-case response time for Baseline (BL), Meta-Cure (MC), and Asymmetric Programming (AP) with different (**a**) Percentages of read operations (**b**) Ratios of random accesses.

As shown in Figure 16, compared with the baseline scheme, there will be some overhead induced by the proposed scheme (on average 3.32%), but the worst case response time is still much smaller than that of Meta-Cure method, which induces 9.33% on average overhead compared with that of the baseline scheme. As for Meta-Cure, when the redundant write operation for every critical page or selective copy operation occurs during garbage collection process, the write operation time will be greatly prolonged and this time will be taken as the worst-case response time.

Compared with the baseline scheme, which does not have the request feedback operation, the worst-case response time of Asymmetric Programming is a little longer than that of the baseline scheme. If the request feedback operation is conducted multiple times, the garbage collection process will be prolonged and that will induce some overhead for the worst case response time.

Figure 16 presents the worst-case response time of Asymmetric Programming with different percentages of read operations and different ratios of random accesses. For our Asymmetric Programming scheme, the request with worst-case response time takes place when the request queue scheduler delays the current write request to serve the next request (as shown in Algorithm 1) and the garbage collection process is triggered at the same time. This request feedback operation may be conducted several times. Thus, the worst-case response time would not be constant with different workloads.

## 7. Conclusions

In this paper, we have proposed Asymmetric Programming, a reliability aware metadata allocation strategy for MLC NAND flash memory storage systems. Asymmetric Programming utilizes the characteristics of the shared pages in MLC NAND flash memory and only stores metadata pages in MSB pages. Asymmetric Programming enhances the reliability of metadata using two strategies: MSB allocation and asymmetric garbage collection. By adopting these two strategies, the error rate is reduced and the utilization of physical blocks is improved. We have conducted experiments on a real hardware platform with ARM Cortex A9 processors and multiple NAND flash memory chips. The results show that our Asymmetric Programming scheme can significantly reduce uncorrectable page errors, without incurring any space overhead. In the future, we plan to apply our strategy to hybrid storage systems with both SLC and MLC NAND flash or with other types of nonvolatile memory. We also plan to further add the issue of the performance variation between MSB and LSB page programming latencies [41,42] into our metadata protection scheme to further improve the reliability of the metadata in MLC NAND flash.

## Figures and Tables

**Table 1:** Quantized uncorrectable page error rate and corresponding RBER (Raw Bit Error Rate).

RBER	2.00 × 10^−6^	4.00 × 10^−6^	6.00 × 10^−6^	8.00 × 10^−6^	2.00 × 10^−5^	4.00 × 10^−5^	6.00 × 10^−5^	8.00 × 10^−5^
LSB pages with 3-bit ECC	3.66 × 10^−9^	5.78 × 10^−8^	2.89 × 10^−7^	8.99 × 10^−7^	3.23 × 10^−5^	4.51 × 10^−4^	2.00 × 10^−3^	5.50 × 10^−3^
MSB pages with 3-bit ECC	2.30 × 10^−10^	3.66 × 10^−9^	1.84 × 10^−8^	5.78 × 10^−8^	2.17 × 10^−6^	3.23 × 10^−5^	1.53 × 10^−4^	4.51 × 10^−4^

**Table 2:** Configuration of the Micron 29F64GCBAAA NAND flash memory

**Configuration**		
Organization	Page Size	8192 + 448 bytes
	Block Size	256 pages
	Plane Size	2 planes
	Device Size	4096 blocks

Performance	Read Page	75 us (MAX)
	Program Page	1300 us (TYP)
	Erase Block	3.8 ms (TYP)

Reliability	Data Retention	JESD47G compliant
	Endurance	3000 P/E cycles

**Table 3:** The characteristic of the workload and the status of Micron 29F64GCBAAA NAND flash memory of Baseline, Meta-Cure, and Asymmetric Programming (BL: Base Line; MC: Meta-Cure; AP: Asymmetric Programming).

**Scheme**	**Workload Characteristic**			**NAND Flash Package status**	
	
	**Number of Requests**	**Percentage of Write Operations**	**Average Request Size**	**Level of Wear (P/E Cycles)**	**ECC Strength**
BL	282,710	93.34%	16.09 KB	2,000	4-bit
MC	282,802	93.19%	16.09 KB	2,000	4-bit
AP	282,464	93.23%	16.09 KB	2,000	4-bit

**Table 4:** The experimental results of Baseline, Meta-Cure, and Asymmetric Programming with Zynq on-board sensor system (BL: Base Line; MC: Meta-Cure; AP: Asymmetric Programming).

**Scheme**	**Uncorrectable Page Errors**		**I/O Performance**		**Garbage Collection**	
		
	**# of Uncorrectable Page Errors**	**Reduction (%) (*vs.* BL)**	**Average Response Time (ns)**	**Overhead (%) (*vs.* BL)**	**# of Block Erase Counts**	**Overhead (%) (*vs.* BL)**
BL	2503	—	2,495,552	—	1,745	—
MC	983	60.70	2,727,154	9.28	1,866	6.93
AP	201	91.97	2,496,564	0.04	1,749	0.23

**Table 5:** The number of uncorrectable page errors for Baseline, Meta-Cure, and Asymmetric Programming with different ECC strengths with a P/E cycle of 1000 and a P/E cycle of 3000 (BL: Base Line; MC: Meta-Cure; AP: Asymmetric Programming).

**ECC Strength**	**1000 P/E Cycles**					**3000 P/E Cycles**				
	
	**# of Uncorrectable Page Errors**			**Reduction (%)**		**# of Uncorrectable Page Errors**			**Reduction (%)**	
	**BL**	**MC**	**AP**	**AP *vs.* BL**	**AP *vs.* MC**	**BL**	**MC**	**AP**	**AP *vs.* BL**	**AP *vs.* MC**
0-bit	9647	6572	5716	40.75	13.04	12,947	12,937	8133	37.18	37.13
1-bit	6270	3589	1384	77.93	61.43	8238	7197	3208	61.06	55.43
2-bit	2559	1002	295	88.47	70.56	4667	4177	1243	73.34	70.24
3-bit	1328	361	57	95.71	84.21	2331	1230	532	77.18	56.75
4-bit	658	135	11	98.33	91.85	1106	570	170	84.63	70.18
5-bit	316	53	3	99.05	94.34	539	141	53	90.17	62.41
6-bit	165	27	2	98.79	92.59	250	80	15	94.00	76.67
7-bit	66	7	2	96.97	71.43	120	30	7	94.17	76.67
8-bit	66	7	2	96.97	71.43	32	4	4	87.50	0.00
Average				88.11	72.32				77.69	56.67

**Table 6:** The I/O performance for Baseline, Meta-Cure, and Asymmetric Programming with different radios of random accesses (BL: Base Line; MC: Meta-Cure; AP: Asymmetric Programming).

**% of Random Accesses**	**Total Requests**	**Average Response Time (ns)**			**Overhead (%)**		**Improvement (%)**

		**BL**	**MC**	**AP**	**MC *vs.* BL**	**AP *vs.* BL**	**AP *vs.*MC**
0	146,443	1,638,774	1,735,443	1,635,948	6.21	0.12	6.09
25	146,381	1,750,170	1,867,718	1,751,090	6.72	0.05	6.66
50	146,353	1,754,543	1,871,834	1,755,464	6.68	0.05	6.64
75	151,467	1,751,297	1,868,359	1,754,623	6.68	0.12	6.56
100	146,355	1,753,841	1,874,425	1,753,383	6.88	0.002	6.90
Average					6.63	0.07	6.57

**Table 7:** The I/O performance for Baseline, Meta-Cure, and Asymmetric Programming with different percentages of read operations (BL: Base Line; MC: Meta-Cure; AP: Asymmetric Programming).

**% of Read Operations**	**Total Requests**	**Average Response Time (ns)**			**Overhead (%)**		**Improvement (%)**

		**BL**	**MC**	**AP**	**MC *vs.* BL**	**AP *vs.* BL**	**AP *vs.* MC**
0	155,704	2,438,562	2,530,800	2,442,056	3.78	0.14	3.64
25	151,518	2,089,008	2,187,306	2,095,965	4.71	0.33	4.38
50	146,353	1,755,354	1,871,839	1,758,980	6.64	0.21	6.43
75	151,477	1,406,386	1,545,373	1,410,152	9.88	0.27	9.61
100	151,442	1,112,380	1,271,339	1,110,935	14.29	0.06	14.23
Average					7.81	0.20	7.61

## References

[1] Wu C.H., Kuo T.W., Chang L.P. (2006). The Design of efficient initialization and crash recovery for log-based file systems over flash memory. ACM Trans. Storage.

[2] Xiang X., Yue L., Liu Z., Wei P. (2008). A ReliableB-Tree Implementation over Flash Memory.

[3] Mylavarapu S.K., Choudhuri S., Shrivastava A., Lee J., Givargis T. FSAF: File system aware flash translation layer for NAND flash memories.

[4] Wu C.H., Lin H.H. (2012). Timing analysis of system initialization and crash recovery for a segment-based flash translation layer. ACM Trans. Des. Autom. Electron. Syst..

[5] Wu C.H., Lin H.H., Kuo T.W. (2010). An adaptive flash translation layer for high-performance storage systems. IEEE Trans. Comput. Aided Des. Integrated Circuits Syst..

[6] Wang Y., Liu D., Qin Z., Shao Z. An endurance-enhanced Flash Translation Layer via reuse for NAND flash memory storage systems.

[7] Park D., Debnath B., Du D. CFTL: A convertible flash translation layer adaptive to data access patterns.

[8] Wang Y., Bathen L.A.D., Shao Z., Dutt N.D. 3D-FlashMap: A physical-location-aware block mapping strategy for 3D NAND flash memory.

[9] Sun G., Kursun E., Rivers J.A., Xie Y. (2013). Exploring the vulnerability of CMPs to soft errors with 3D stacked nonvolatile memory. ACM J. Emerg. Technol. Comput. Syst..

[10] Zhang C., Wang Y., Wang T., Chen R., Liu D., Shao Z. (2014). Deterministic Crash Recovery for NAND Flash Based Storage Systems.

[11] Miranda A., Effert S., Kang Y., Miller E.L., Brinkmann A., Cortes T. Reliable and randomized data distribution strategies for large scale storage systems.

[12] Shi L., Qiu K., Zhao M., Xue C.J. (2014). Error Model Guided Joint Performance and Endurance Optimization for Flash Memory. IEEE Trans. Comput. Aided Des. Integrated Circuits Syst..

[13] Guo J., Chen Z., Wang D., Shao Z., Chen Y. DPA: A data pattern aware error prevention technique for NAND flash lifetime extension.

[14] Guo J., Yang J., Zhang Y., Chen Y. Low cost power failure protection for MLC NAND flash storage systems with PRAM/DRAM hybrid buffer.

[15] Agrawal N., Bolosky W.J., Douceur J.R., Lorch J.R. (2007). A five-year study of file-system metadata. ACM Trans. Storage.

[16] Chen J., Wei Q., Chen C., Wu L. FSMAC: A file system metadata accelerator with non-volatile memory.

[17] Wang Y., Bathen L.A.D., Dutt N.D., Shao Z. Meta-Cure: A reliability enhancement strategy for metadata in NAND flash memory storage systems.

[18] Zhou R., Liu M., Li T. Characterizing the efficiency of data deduplication for big data storage management.

[19] Pan Y., Dong G., Zhang T. (2013). Error rate-based wear-leveling for NAND flash memory at highly scaled technology nodes. IEEE Trans. Very Large Scale Integration (VLSI) Syst..

[20] Takeuchi K., Tanaka T., Tanzawa T. (1998). A multipage cell architecture for high-speed programming multilevel NAND flash memories. IEEE J. Solid State Circuits.

[21] Vallina F.M., Kohn C., Joshi P. Zynq All Programmable SoC Sobel Filter Implementation Using the Vivado HLS Tool. http://china.zylinks.com/support/documentation/application_notes/xapp890-zynq-sobel-vivado-hls.pdf.

[22] Venkateswaran S. (2008). Essential Linux Device Drivers.

[23] Wang C., Wong W.F. TreeFTL: Efficient RAM management for high performance of NAND flash-based storage systems.

[24] Liu D., Wang Y., Qin Z., Shao Z., Guan Y. A (2012). Space Reuse Strategy for Flash Translation Layers in SLC NAND Flash Memory Storage Systems. IEEE Trans. Very Large Scale Integration (VLSI) Syst..

[25] Guan Y., Wang G., Wang Y., Chen R., Shao Z. (2013). BLog: Block-Level Log-Block Management for NAND Flash Memorystorage Systems. Proceedings of ACM SIGPLAN Notices.

[26] Qin Z., Wang Y., Liu D., Shao Z. Real-time flash translation layer for NAND flash memory storage systems.

[27] Chang L.P., Wen C.Y. (2014). Reducing asynchrony in channel garbage-collection for improving internal parallelism of multichannel solid-state disks. ACM Trans. Embedded Comput. Syst..

[28] Wu C.H. A, Bad-Block Test (2012). Design for Multiple Flash-Memory Chips. J. Inf. Sci. Eng..

[29] Chang L.P., Chou T.Y., Huang L.C. (2013). An adaptive, low-cost wear-leveling algorithm for multichannel solid-state disks. ACM Trans. Embedded Comput. Syst..

[30] Qin Z., Wang Y., Liu D., Shao Z., Guan Y. (2011). MNFTL: An efficient flash translation layer for MLC NAND flash memory storage systems.

[31] Woodhouse D. JFFS: The journalling flash file system.

[32] Manning C. How YAFFS Works. http://users.actrix.co.nz/manningc/yaffs-docs/HowYaffsWorks.pdf.

[33] Huebner E., Bem D., Wee C.K. (2006). Data hiding in the NTFS file system. Digit. Investig.

[34] Zhang Z., Zhang M. (2005). Analysis of FAT32 File System. Comput. Digit. Eng..

[35] Preslan K.W., Barry A.P., Brassow J.E., Erickson G.M., Nygaard E., Sabol C.J., Soltis S.R., Teigland D.C., O'Keefe M.T. A 64-bit, shared disk file system for Linux.

[36] Long C., Xiong J., Liu Y. Techniques of power-gating to kill sub-threshold leakage.

[37] Mielke N., Marquart T., Ning W., Kessenich J., Belgal H., Schares E., Trivedi F., Goodness E., Nevill L.R. Bit error rate in NAND Flash memories.

[38] Agarwal R., Marrow M. A closed-form expression for write amplification in NAND Flash.

[39] Levine D.D. (1998). Iometer User's Guide. Intel Server Architecture Lab..

[40] Xilinx 7 Series FPGAs and Zynq-7000 All Programmable SoC XADC Dual 12-Bit 1 MSPS Analog-to-Digital Converter User Guide. http://www.xilinx.com/support/documentation/user_guides/ug480_7Series_XADC.pdf.

[41] Jung M., Wilson E.H., Donofrio D., Shalf J., Kandemir M.T. NANDFlashSim: Intrinsic latency variation aware NAND flash memory system modeling and simulation at microarchitecture level.

[42] Grupp L.M., Caulfield A.M., Coburn J., Swanson S., Yaakobi E., Siegel P.H., Wolf J.K. Characterizing flash memory: Anomalies, observations, and applications.

